# Comprehensive Profile of Acute Mitochondrial Dysfunction in a Preclinical Model of Severe Penetrating TBI

**DOI:** 10.3389/fneur.2019.00605

**Published:** 2019-06-11

**Authors:** Jignesh D. Pandya, Lai Yee Leung, Xiaofang Yang, William J. Flerlage, Janice S. Gilsdorf, Ying Deng-Bryant, Deborah A. Shear

**Affiliations:** ^1^Brain Trauma Neuroprotection Branch, Center for Military Psychiatry and Neuroscience, Walter Reed Army Institute of Research, Silver Spring, MD, United States; ^2^Department of Surgery, Uniformed Services University of the Health Sciences, Bethesda, MD, United States

**Keywords:** traumatic brain injury (TBI), penetrating ballistic-like brain injury (PBBI), alternative biofuels, brain energy metabolism, energy crisis, mitochondria preferred substrates, dehydrogenase activities, therapeutics

## Abstract

Mitochondria constitute a central role in brain energy metabolism, and play a pivotal role in the development of secondary pathophysiology and subsequent neuronal cell death following traumatic brain injury (TBI). Under normal circumstances, the brain consumes glucose as the preferred energy source for adenosine triphosphate (ATP) production over ketones. To understand the comprehensive picture of substrate-specific mitochondrial bioenergetics responses following TBI, adult male rats were subjected to either 10% unilateral penetrating ballistic-like brain injury (PBBI) or sham craniectomy (*n* = 5 animals per group). At 24 h post-injury, mitochondria were isolated from pooled brain regions (frontal cortex and striatum) of the ipsilateral hemisphere. Mitochondrial bioenergetics parameters were measured *ex vivo* in the presence of four sets of metabolic substrates: pyruvate+malate (PM), glutamate+malate (GM), succinate (Succ), and β-hydroxybutyrate+malate (BHBM). Additionally, mitochondrial matrix dehydrogenase activities [i.e., pyruvate dehydrogenase complex (PDHC), alpha-ketoglutarate dehydrogenase complex (α-KGDHC), and glutamate dehydrogenase (GDH)] and mitochondrial membrane-bound dehydrogenase activities [i.e., electron transport chain (ETC) Complex I, II, and IV] were compared between PBBI and sham groups. Furthermore, mitochondrial coenzyme contents, including NAD_(t)_ and FAD_(t)_, were quantitatively measured in both groups. Collectively, PBBI led to an overall significant decline in the ATP synthesis rates (43–50%; ^*^p < 0.05 vs. sham) when measured using each of the four sets of substrates. The PDHC and GDH activities were significantly reduced in the PBBI group (42–53%; ^*^p < 0.05 vs. sham), whereas no significant differences were noted in α-KGDHC activity between groups. Both Complex I and Complex IV activities were significantly reduced following PBBI (47–81%; ^*^p < 0.05 vs. sham), whereas, Complex II activity was comparable between groups. The NAD_(t)_ and FAD_(t)_ contents were significantly decreased in the PBBI group (27–35%; ^*^p < 0.05 vs. sham). The decreased ATP synthesis rates may be due to the significant reductions in brain mitochondrial dehydrogenase activities and coenzyme contents observed acutely following PBBI. These results provide a basis for the use of “alternative biofuels” for achieving higher ATP production following severe penetrating brain trauma.

## Highlights

- Metabolic substrates mediated decline in ATP synthesis following TBI.
- Decreased brain mitochondrial dehydrogenase activities following TBI.
- Decreased brain mitochondrial NAD_(t)_ and FAD_(t)_ contents following TBI.
- Alternative biofuels are recommended to overcome energy crisis after TBI.

## Introduction

There is a growing interest in further understanding of the brain metabolic responses following traumatic brain injury (TBI) to facilitate acute critical care management of TBI patients; and develop novel targeted therapeutic interventions for TBI. TBI produces immediate hypo- or hyper-metabolic responses, which are indicated by the changes in the cerebral metabolic rate of glucose (CMRglc), blood flow and oxygenation. Although the magnitude and duration of CMRglc metabolic pattern varies with the injury severity, species, and the type of brain injury models, all studies consistently showed transient hyper-function followed by prolonged depression during acute to sub-acute phase of secondary injury in both pre-clinical injury models and in TBI patients (1–4) This metabolic shift has been observed in different experimental injury models and clinically over the acute and sub-acute phases following TBI (5–8). During this period, concomitant increases in anaerobic lactate accumulation due to glycolysis was evident in both tissue and extracellular space (3, 5, 9–12). Therefore, a decline in brain energy resulting from TBI is a significant issue that remains to be addressed in the management of TBI (13). In fact, several clinical reviews have recommended early intervention of supplying energy intermediate substrates for improvements in mortality and neurological outcomes following TBI (13–15).

Mitochondria play a pivotal role in maintaining cellular energy homeostasis critical to neuronal cell survival. Previous studies have implicated mitochondrial dysfunction as a prominent feature of TBI. Abnormal mitochondrial functions include reduced respiratory rates, depleted energy stores (i.e., adenosine triphosphate, ATP), increased free radical production, mitochondrial calcium overload, and early opening of mitochondrial permeability transition pore (mPTP) (16–18). The increased production of free radicals (i.e., reactive oxygen and nitrogen species, ROS/RNS) overwhelms endogenous antioxidants resulting in oxidative stress (19, 20). Additionally, mitochondria release many pro-inflammatory, apoptotic and necrotic signaling factors contributing to cell death overtime (17, 21–23).

In the current study, we hypothesized that TBI leads to altered utilization of glucose and ketone intermediates for brain energy production. We compared mitochondrial ATP synthesis rates for glucose and ketone intermediate substrates (i.e., pyruvate, glutamate, succinate, and β-hydroxybutyrate), each using an identical *ex vivo* condition. Additionally, mitochondrial dehydrogenase activities, and coenzyme contents were quantified following PBBI vs. sham. Collectively, our results provide a basis for the use of therapeutic drugs and nutraceuticals as “alternative biofuels” for the management of energy crisis following brain trauma.

## Materials and Methods

### Materials

Mannitol, sucrose, bovine serum albumin (BSA), N-2-hydroxyethylpiperazine-N′-2-ethanesulfonic acid (HEPES) potassium salt, potassium phosphate monobasic anhydrous (KH_2_PO_4_), magnesium chloride (MgCl_2_), ethylene-diamine-tetra-acetic acid (EDTA), ethylene-glycol-tetra-acetic acid (EGTA), pyruvate, malate, glutamate, succinate, β-hydroxybutyrate, α-ketoglutarate, adenosine-5′-diphosphate (ADP), oligomycin A, carbonyl cyanide 4-(trifluoromethoxy)phenylhydrazone (FCCP), rotenone, succinate, dimethyl sulfoxide (DMSO) were purchased from Sigma-Aldrich (St. Louis, MO, USA). BCA protein assay kit was purchased from Pierce (Rockford, IL). Both NAD_(t)_ and FAD_(t)_ content assessments kits were purchased from Sigma-Aldrich (St. Louis, MO, USA). Mitochondrial substrates and inhibitors stock solutions were prepared and aliquots stored at −80°C.

### Animals

Adult male Sprague-Dawley rats (280–350 g, Charles River Laboratories, Raleigh, VA) were used for this study. Animals were housed individually under a normal 12 h light/dark cycle (lights on at 0600 EST) in a well-ventilated facility accredited by the Association for Assessment and Accreditation of Laboratory Animal Care (AAALAC), and allowed 7 d for acclimation to the housing facility before any procedures were performed. All experimental procedures were approved by the Institutional Animal Care and Use Committee (IACUC) at Walter Reed Army Institute of Research (WRAIR). Animal studies were conducted in compliance with the Animal Welfare Act, the Guide for the Care and Use of Laboratory Animals (National Research Council), and other federal statutes and regulations relating to animals and experiments involving animals.

### Penetrating Ballistic-Like Brain Injury (PBBI) Model

All surgical procedures were performed under isoflurane anesthesia (3–5% for induction and 2% for maintenance) and aseptic conditions with careful monitoring of physiological vital signs. The PBBI surgery was performed as described previously (24–26). The PBBI apparatus consists of a specifically designed probe (Kadence Science, Lake Success, NY), a stereotaxic frame (Kopf, Tujunga, CA) and a hydraulic pressure-pulse generator (4B080; MITRE, MA). The probe was made of a 20G stainless steel tube with fixed perforations along one end which was sealed by a piece of airtight elastic tubing. The probe was secured on the probe holder with the un-perforated end attached to the pulse generator, angled at 50° from the vertical axis and 25° counter clockwise from the midline. Core body temperature was maintained normothermic (~37°C) using a heating blanket (Harvard Apparatus, South Natick, MA). Under isoflurane anesthesia (2%; in air/oxygen mixture), rat head was secured in the stereotaxic device for insertion of the PBBI probe. After a midline scalp incision, a right frontal cranial window (diameter = 4 mm) was created using a dental drill to expose the right frontal pole (+4.5 mm AP, +2 mm ML to bregma). The PBBI probe was then advanced through the cranial window into the right hemisphere to a depth of 1.2 cm from the surface of the brain. Once the probe was in place, the pulse generator was activated by a computer to release a pressure pulse calibrated to produce a rapid expansion of the water-filled elastic tubing to create an elliptical shaped balloon (diameter = 0.633 mm) to a volume equal to 10% of the total brain volume. This rapid inflation/deflation (duration = 40 ms) produced a temporary cavity in the brain. After deflation, the probe was immediately retracted from the brain and the cranial opening was sealed with sterile bone wax, and the skin incision was closed with wound clips. All sham animals underwent craniectomy only with no insertion of the PBBI probe.

### Mitochondrial Isolation

At 24 h post-PBBI, animals were asphyxiated with CO_2_, rapidly decapitated, and the brains were removed and placed in mitochondrial isolation buffer at 4°C. From ipsilateral hemisphere of PBBI and sham injured brains, the frontal cortex and striatum regions were isolated and pooled together for mitochondrial isolation. The pooled regions represent the injury core and perilesional area of the injury, where extensive cell death may occur due to the initial mechanical force (primary injury) and excitotoxicity (secondary injury). Mitochondria were isolated under identical experimental conditions using an established Ficoll-based mitochondrial ultra-purification procedure (27–29). All isolation steps were performed at 4°C using mitochondrial isolation buffer (MIB) composed of 215 mM Mannitol, 75 mM Sucrose, 0.1% BSA, 1 mM EGTA, and 20 mM HEPES at pH 7.2. Tissue homogenates were centrifuged at 1,300 × g for 3 min to remove cell debris and nuclei, and collected supernatants were then centrifuged at 13,000 × g for 10 min to pellet crude mitochondria. The resultant crude mitochondrial samples were re-suspended in 500 μl of MIB and kept in a nitrogen cell decompression bomb (model 4,639, Parr Instrument Co., Moline, IL, USA) at 1,200 psi for 10 min at 4°C to release mitochondria from synaptosomes. The mitochondrial samples were then placed on top of a discontinuous Ficoll gradient (layered 2 ml of 7.5% Ficoll solution on top of 2 ml of 10% Ficoll solution) and centrifuged at 100,000 × g for 30 min at 4°C using a Beckman ultracentrifuge with SW55Ti rotor (Beckman Coulter, IN, USA). Ultrapure mitochondrial pellets formed at the bottom were carefully collected, avoiding contamination from ruptured synaptic plasma membranes isolated at the interface. These pure mitochondrial pellets were resuspended in 2 ml MIB^−^ (without 1 mM EGTA buffer) and centrifuged at 10,000 × g for 10 min. The washed mitochondrial pellets were finally re-suspended in MIB^−^ to achieve desirable mitochondrial protein concentration (~10 mg/ml) for mitochondrial function assessments. Mitochondrial protein concentration was determined using the BCA protein assay kit measuring absorbance at 562 nm with BioTek Synergy HT plate reader (Winooski, VT, USA). Following the completion of mitochondrial isolation, mitochondrial respiration was measured within ~3 h post-isolation; whereas remaining samples were stored at −80°C for dehydrogenase activities and coenzyme content assessments.

### Mitochondrial Respiration

The real-time mitochondrial respiration was assessed by the Clark-type oxygen electrode in a continuously stirred, sealed chamber thermostatically maintained at 37°C (Oxytherm System, Hansatech Instruments Ltd.,), as described previously (16, 27, 29). Approximately 50 μg of mitochondrial protein was added into the chamber containing 250 μl of KCl-based respiration buffer (125 mM KCl, 2 mM MgCl_2_, 2.5 mM KH_2_PO_4_, 0.1% BSA, 20 mM HEPES, pH 7.2). After 1 min equilibration, the State II respiration was initiated by the addition of either one of the four separate sets of metabolic substrates [e.g., pyruvate+malate (PM), glutamate+malate (GM), succinate (Succ), or β-hydroxybutyrate+malate (BHBM)] in the respiration chamber. The State III respiration rate was measured subsequently after addition of two boluses of 150 μM ADP followed by State IV respiration rates that were measured by the addition of 1 μM oligomycin. The mitochondrial uncoupler FCCP (1 μM) was added at last to measure uncoupling respiration rates (State V) in the chamber. For each mitochondrial sample, all four substrates driven mitochondrial respiration were individually performed; and their respiration rates (i.e., from State II to State V) were recorded to calculate oxygen consumption rates (nmols O_2_ consumed/mg protein).

### Mitochondrial Dehydrogenase Activity Assessments

All remaining −80°C stored mitochondrial samples were freeze-thawed and sonicated together three times before measuring enzyme activities at 37°C. Mitochondrial matrix dehydrogenase activities [i.e., pyruvate dehydrogenase complex (PDHC), alpha-ketoglutarate dehydrogenase complex (α-KGDHC), and glutamate dehydrogenase (GDH)] and membrane-bound dehydrogenase activities (i.e., electron transport chain (ETC) Complex I, II, and IV) were performed by either absorbance or florescence based assays using an automated 96-well microplate reader (BioTek Instruments, INC, Winooski, VT, USA) as described below (27, 30–34).

### Pyruvate Dehydrogenase Complex (PDHC) Enzyme Activity

The mitochondrial gate keeper enzyme PDHC catalyzes the oxidative decarboxylation of pyruvate to acetyl-CoA and thereby links the glycolysis pathway to TCA cycle. It provides intermediate acetyl-CoA substrates to TCA cycle for energy metabolism. The first subunit of the PDHC i.e., pyruvate dehydrogenase (PDH) enzyme activity was measured using previously described methods with slight modification. The assay was performed using a substrates, inhibitor and cofactor mixtures in a buffer containing 50 mM KCl, 10 mM HEPES pH 7.4, 0.3 mM thiamine pyrophosphate (TPP), 10 μM CaCl_2_, 0.2 mM MgCl_2_, 5 mM pyruvate, 1 μM rotenone, and 0.2 mM NAD. Ficoll-purified mitochondrial protein (8 μg / well) was assayed in triplicate for each sample. The enzyme reaction was started by the addition of 0.14 mM coenzyme A. The fluorescence based enzyme activity assay was performed (Ex λ 340 nm, Em λ 460 nm), and increment of NADH fluorescence was measured per minute interval. The PDHC activity was calculated and expressed as % change in PBBI vs. sham.

### Glutamate Dehydrogenase (GDH) Enzyme Activity

The mitochondrial enzyme GDH catalyzes the reversible oxidative deamination of glutamate to α-ketoglutarate and serves as a key link between anabolic and catabolic pathways. The GDH enzyme activity (10 μg/well) assay was determined (i.e., absorbance 450 nm) by a coupled enzyme colorimetric assay (100 μl assay volume) in which glutamate was consumed by GDH generating NADH, which in turn reacts with a probe proportional to the GDH activity present. One unit of GDH is the amount of enzyme that generates 1 mmols of NADH per minute at pH 7.6 at 37°C. The GDH assay was performed using commercially available assay kit (Sigma-Aldrich, USA) and the data was expressed as % change in PBBI vs. sham.

### α-Ketoglutarate Dehydrogenase Complex (α-KGDHC) Enzyme Activity

The first subunit form of the mitochondrial enzyme α-KGDHC, 2-oxoglutarate dehydrogenase complex catalyzes the conversion of 2-oxoglutarate to succinyl-CoA and CO_2_ in the TCA cycle. The first subunit of the α-KGDHC i.e., α-ketoglutarate dehydrogenase (α-KGDH) activity (10 μg/well) assay was determined (i.e., absorbance 450 nm) by a coupled enzyme colorimetric assay (100 μl assay volume) in which 2-oxoglutarate was consumed by α-KGDH generating NADH, which in turn reacts with a probe proportional to the α-KGDH activity present. One unit of α-ketoglutarate dehydrogenase activity is the amount of enzyme that generates 1 mmols of NADH per minute at pH 7.5 at 37°C. The α-KGDH assay was performed by commercially available assay kit (Sigma-Aldrich, USA) and the data was expressed as % change in PBBI vs. sham.

### Complex I (NADH Dehydrogenase) Enzyme Activity

Complex I, or NADH dehydrogenase, is the first enzyme of the mitochondrial ETC. It catalyzes the transfer of electrons from NADH to coenzyme Q. The Complex I activity assay was performed in 25 mM KH_2_PO_4_ buffer (pH 7.2) containing 5 mM MgCl_2_, 1 mM KCN, 1 mg/ml BSA and 150 μM NADH. Mitochondrial protein (6 μg/well) was added to the reaction buffer, and the assay was performed in the absence or presence of rotenone (10 μM) to determine rotenone-insensitive and rotenone-sensitive enzyme activities. The reaction was started by the addition 50 μM of coenzyme Q_1_. The fluorescence based enzyme activity assay was performed (Ex λ 340 nm, Em λ 460 nm), and decrease in fluorescence of NADH was measured per 1 min intervals. The Complex I activity was calculated and expressed as % change in PBBI vs. sham.

### Complex II (Succinate Dehydrogenase) Enzyme Activity

Complex II, or succinate dehydrogenase, is the only membrane-bound enzyme of the TCA cycle that participates additionally in the ETC activities. The FAD containing enzyme catalyzes the oxidation of succinate to fumarate with the reduction of ubiquinone to ubiquinol. The enzyme activity was determined by coupled reactions measuring the change in absorbance of a dye, 2,6-Dichlorophenolindophenol (DCIP), at 600 nm using a BioTek Synergy HT plate reader (BioTek, Winooski, VT, USA). Briefly, the mitochondrial protein (8 μg/well) was added to 200 mM KH_2_PO_4_ (pH 7.0) assay buffer containing 20 mM K-succinate as substrate for Complex II, 10 μM EDTA, 0.01% Triton X-100, 1 μg coenzyme Q_10_ in a total assay volume of 100 μl/well. The Complex I inhibitor rotenone was not included in the assay system. The 30 μl of concentrated DCIP dye (20 mg%) was added to each well, giving initial absorbance readings near 0.9–1.0. In the reaction, succinate is oxidized to fumarate and reduces DCIP dye (blue color) into DCIPH_2_ (colorless) compound. During this succinate-DCIP oxidation-reduction, coenzyme Q_10_ acts as an intermediate compound that transiently accepts electrons and further donates to DCIP. The decrease in DCIP absorbance was measured per minute based on nmols of succinate oxidized; and expressed as % change in PBBI vs. sham.

### Complex IV (Cytochrome C Oxidase) Enzyme Activity

Complex IV is the terminal electron acceptor enzyme in the mitochondrial ETC complexes. It receives an electron from four cytochrome c molecules and transfers them to one oxygen molecule to convert molecular oxygen; together with translocating four protons into intermembrane space from mitochondrial matrix to establish a transmembrane electrochemical potential (ΔΨm). The cytochrome c oxidase activity was measured in 10 mM KH_2_PO_4_ buffer by co-incubating mitochondrial protein (6 μg/well). The reaction was initiated by adding 50 μM reduced cytochrome c. The rate of oxidation of cytochrome c was measured by detecting decrease in absorbance of reduced cytochrome c at 550 nm per 1 min intervals. The Complex IV activity was calculated and expressed as % change in PBBI vs. sham.

### Mitochondrial Coenzyme Contents

#### NAD_(t)_ Quantification

Nicotinamide adenine dinucleotide (NAD) is an enzymatic cofactor involved in many redox reactions. NAD functions as an electron carrier, cycling between the oxidized (NAD) and reduced (NADH) forms. In addition to its role in redox reactions, NAD plays critical roles in several cellular metabolic reactions. In a colorimetric assay (i.e., absorbance 450 nm), the total form of NAD or NAD_(t)_ (i.e., the combined form of both NAD and NADH) was quantified using commercially available NAD quantification kit (Sigma-Aldrich, USA). This assay is specific for NAD_(t)_ and does not detect NADP nor NADPH. Briefly, mitochondrial proteins (100 μg) were re-suspended in 100 μl of NAD extraction buffer. Samples were vortexed and freeze-thawed three times, and any remaining insoluble debris was finally pelleted at 13,000 × g for 10 min and discarded. The extracted supernatant was transferred into a new tube and stored for measurement (50 μg/well). The NAD_(t)_ concentration was measured as pmoles per mg protein based on a standard curve and expressed as % change in PBBI vs. sham.

#### FAD_(t)_ Quantification

Flavin Adenine Dinucleotide (FAD) is a coenzyme, synthesized from riboflavin, which plays critical roles in many metabolic pathways. FAD functions as an electron carrier in multiple redox reactions, cycling between FAD and FADH_2_. The primary sources of reduced FAD in eukaryotic metabolism are the TCA cycle and the beta oxidation reaction pathways. For total FAD or FAD_(t)_ (i.e., the combined form of both FAD and FADH_2_) content measurement, mitochondrial proteins (100 μg) were re-suspended in 100 μl of FAD extraction buffer and deproteinizes with 8% perchloric acid solution. Briefly vortex and incubate the precipitate mixture for 5 min. The mixture was centrifuged at 1,500 × g for 10 min and then transferred into a new tube for measurement (50 μg/well). The FAD_(t)_ concentration was measured using fluorescence-based assay (Ex λ 535 and Em λ 587 nm) using commercially available FAD quantification kit (Sigma-Aldrich, USA). The FAD_(t)_ concentration were measured as pmoles per mg protein based on a standard curve and expressed as % change in PBBI vs. sham.

### Statistical Analysis

*A priory* power analyses were conducted to determine the sample size within individual experiments using G^*^Power 3 (Germany) statistical program. The power analyses based on previous mitochondrial bioenergetics data indicated that an *n* = 5 sample size/group, is sufficient to detect a 20% change as statistically significant (*p* < 0.05) with power of 0.8 compared to control. The experimental data are presented as bar graphs with error bars represented by ± standard error of the mean (SEM). All mitochondrial samples were prepared from individual animals (*n* = 5/group) using brain tissue derived from the cortex/striatum in the injured hemisphere. All samples were evaluated as triplicates in each experiment. An unpaired *t*-test was used for between-group comparisons (2 groups) whereas multiple group (> 2 groups) comparisons were conducted using analysis of variance (ANOVA) followed by a Fisher protected least squared differences (PLSD) *post-hoc* test (GraphPad Prism 6 software package, GraphPad Software, Inc. La Jolla, CA). Statistical significance was defined at ^*^p < 0.05.

## Results

**Figure 1:**
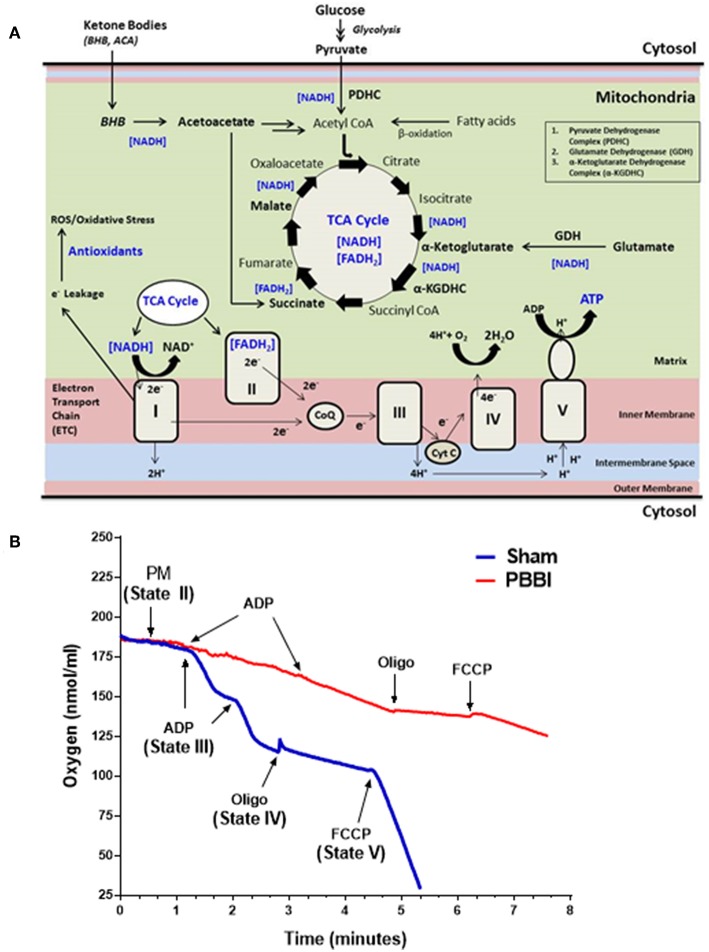
Overview of mitochondrial respiration following PBBI. **(A)** As illustrated in the schematic, brain mitochondrial substrates (i.e., glucose and ketone intermediates) oxidation leads to ATP synthesis through coordinated metabolic reactions of mitochondrial enzymes. Glucose oxidation yield pyruvate through glycolysis, which may further converted to acetyl CoA by enzyme PDHC. Glutamate may oxidized to α-Ketoglutarate by enzyme GDH. Ketone bodies (i.e., BHB or acetoacetate) may metabolized to acetyl CoA or Succinate. All brain energy metabolic substrates (i.e., pyruvate, glutamate, Succ, and BHB) oxidation increases the pool of reducing equivalents (i.e., NADH and FADH_2_); and donates electrons to Complex I or II of ETC enzyme complexes, which triggers the flow of electrons down to the final accepter O_2_ to form H_2_O at Complex IV. Concomitantly, proton-pumping from matrix to the intermembrane space by mitochondrial ETC complexes I, III, and IV generates the mitochondrial membrane potential (ΔΨm). The ΔΨm is then utilized to phosphorylate ADP to ATP through Complex V (i.e., ATP synthase). The complete oxidation of substrates in presence of O_2_ is linked with energy (i.e., ATP synthesis); therefore this process is termed as oxidative phosphorylation (OXPHOS). During OXPHOS, electron leaks (~2% electron leak) from mitochondrial ETC may generate reactive oxygen and nitrogen free radical species (i.e., ROS and RNS, respectively) and subsequent oxidative stress; which are counterbalanced by antioxidants in uninjured brain. **(B)** Representative traces PM-driven mitochondrial respiration in PBBI and sham groups. Briefly, incubate 50 μg of mitochondrial protein into the sealed Clark-type oxygen electrode chamber containing KCl-based respiration buffer (Hansatech Instruments, England). In the real-time condition, sequentially add metabolic substrates/inhibitors of the mitochondrial respiration chain i.e., PM (~1 min), ADP (~2–3 min), oligomycin (~4 min), and FCCP (~6 min) to measure oxygen consumption rates (nmols/ml). The illustrated mitochondrial bioenergetics parameters a.k.a. State II respiration (i.e., basal respiration in presence of PM as the substrate set); State III respiration (ATP synthesis in presence of ADP); State IV respiration (i.e., proton leakage in presence of ETC complex V inhibitor oligomycin); and State V respiration (i.e., uncoupled respiration rates in presence of mitochondrial ETC uncoupler FCCP) were measured for each mitochondrial samples. Similarly, other mitochondrial substrates (i.e., GM, Succ, and BHBM) driven respiration rates were quantified separately. As noted here, the PBBI group (red trace) displayed reduced PM driven ATP synthesis (State III) and uncoupled respiration (State V) compared to sham (blue trace) group.

Mitochondrial bioenergetics parameters were evaluated in brain samples that were pooled from the frontal cortex and striatum of the injured hemisphere. At 24 h post-PBBI, mitochondrial bioenergetics parameters (i.e., State II to State V respiration), mitochondrial dehydrogenase activities, and coenzyme contents were measured in the sham and PBBI groups. The schematic overview of brain mitochondrial metabolic reactions and bioenergetics (Figure 1A), and the real-time tracing of PM-driven mitochondrial respiration in PBBI vs. sham group were represented as illustrated (Figure 1B).

### Global Mitochondrial Bioenergetics Depression in PBBI

**Figure 2:**
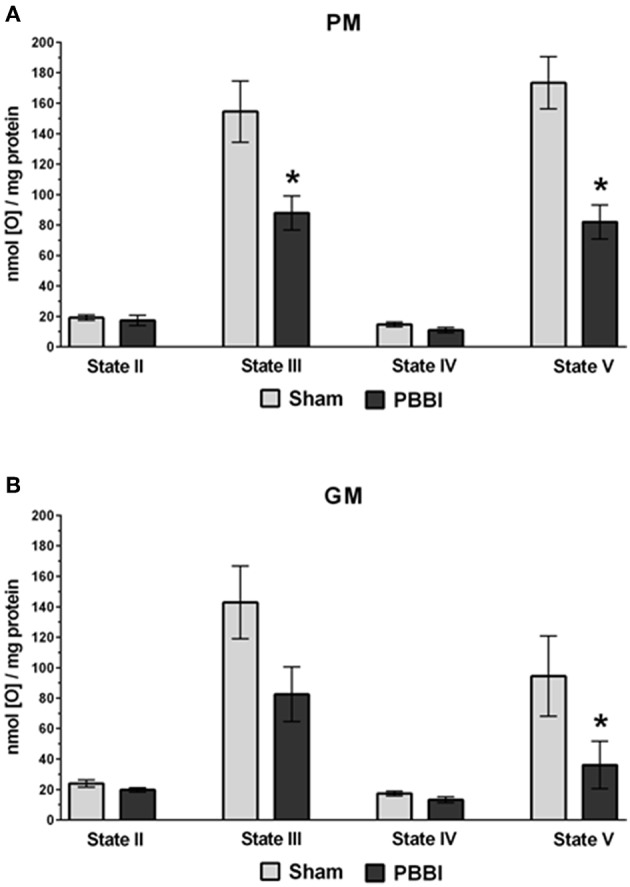
Decreased ATP synthesis in the presence of metabolic substrates PM and GM following PBBI. **(A,B)** At 24 h post-PBBI or sham injury, mitochondrial respiration rates were measured using Clark-type oxygen electrode in the presence of metabolic substrates PM and GM. PBBI reduced ATP synthesis (State III) with PM (43%, ^*^p < 0.05) and GM (42%, *p* = 0.08 non-significant). No change in basal respiration (State II) and proton leaks (State IV) rates were observed. The FCCP induced uncoupling rates (State V) with PM and GM were significantly reduced in the PBBI group (53–62%, ^*^p < 0.05). Bars represent group means ± SEM (*n* = 5 animals / group). ^*^p < 0.05 compared to sham injured group (unpaired *t*-test).

**Figure 3:**
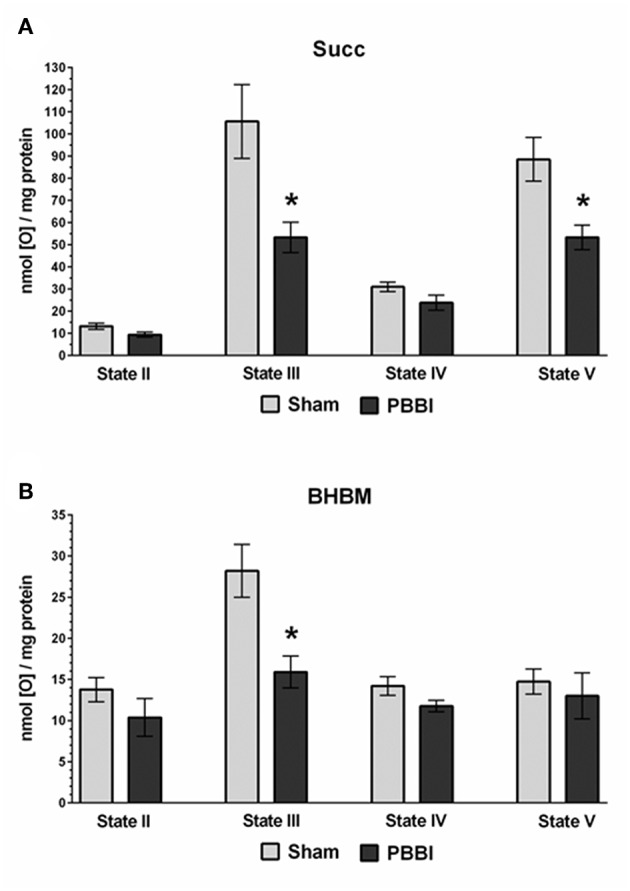
Decreased ATP synthesis in the presence of metabolic substrates Succ and BHBM following PBBI. **(A,B)** At 24 h post-PBBI or sham injury, mitochondrial respiration rates were measured using Clark-type oxygen electrode in the presence of metabolic substrates succinate (Succ) and β-hydroxybutyrate+malate (BHBM). PBBI reduced the Complex II driven ATP synthesis (State III) with Succ (50%, ^*^p < 0.05) and BHBM (43%, ^*^p < 0.05). No change in basal respiration (State II) and proton leaks (State IV) rates were observed. The FCCP induced uncoupling rates (State V) with Succ were significantly reduced in the PBBI group (40%, ^*^p < 0.05). Bars represent group means ± SEM (*n* = 5 animals / group). ^*^p < 0.05 compared to sham injured group (unpaired *t*-test).

At 24 h post-PBBI or sham injury, mitochondrial respiration rates were measured in the presence of glucose intermediate substrate sets: pyruvate+malate (PM) and glutamate+malate (GM) (Figure 2). Following PBBI, mitochondrial respiration with PM showed a significant reduction in both State III (43%, ^*^p < 0.05) and State V (53%, ^*^p < 0.05) respiration. No change in PM driven basal respiration (State II) and proton leaks (State IV) were observed between the PBBI vs. sham groups (Figure 2A). Substrates GM showed similar trend in the PBBI group. Comparing to sham, the mitochondrial respiration with GM showed a trend toward altered State III respiration (42%, *p* = 0.08, non-significant), whereas State V respiration was reduced significantly (62%, ^*^p < 0.05) in the PBBI group. No injury-induced changes were detected in GM driven basal respiration (State II) and proton leaks (State IV) (Figure 2B). Similarly, we evaluated mitochondrial bioenergetics parameters in the presence of two sets of additional glucose and ketone intermediates: succinate (Succ) and β-hydroxybutyrate+malate (BHBM) (Figure 3). When Succ was used as the metabolic substrate, both State III (50%, ^*^p < 0.05) and State V (40%, ^*^p < 0.05) respiration were significantly decreased in PBBI compared to sham. No change in Succ driven basal respiration (State II) and proton leaks (State IV) rates were observed between two groups (Figure 3A). With BHBM as substrates, the State III respiration rate was reduced significantly (43%, ^*^p < 0.05) in PBBI; whereas BHBM driven basal respiration (State II), proton leaks (State IV), and uncoupling rate (State V) were comparable between the sham and PBBI groups (Figure 3B).

### Substrate Preference for ATP Synthesis

**Figure 4:**
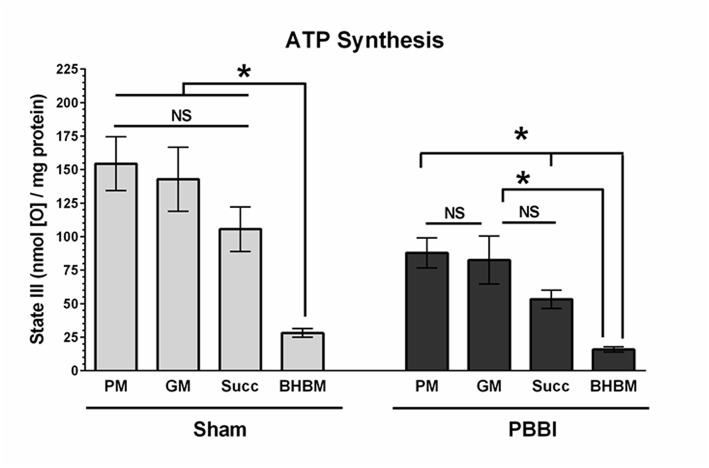
Comparison of ATP synthesis rates with-in the experimental group. The ATP synthesis rates of the four substrates (i.e., PM, GM, Succ, and BHBM) were compared with-in sham or PBBI group. In the sham group, the BHBM showed significantly less ATP synthesis than any other substrates (PM=GM=Succ>BHBM, ^*^p < 0.05). In the PBBI group, the ATP synthesis of PM, Succ, and BHBM were significantly distinct amongst each other (PM = GM>Succ>BHBM, ^*^p < 0.05). Bars represent group means ± SEM (*n* = 5 animals / group). ^*^p < 0.05 compared within four substrates groups of sham or PBBI (ANOVA, Fisher *post-hoc* test).

To evaluate with-in group substrate preferences, the ATP synthesis rates of the four substrates were compared under either sham or PBBI condition (Figure 4). In the sham group, the PM, GM, and Succ substrate driven ATP synthesis (State III) were comparable; whereas the BHBM driven ATP synthesis was significantly lower compared to other substrates (PM = GM = Succ>BHBM, ^*^p < 0.05). In the PBBI group, the pattern of substrate utilization for ATP synthesis remained identical, but at a lower magnitude compared to the sham group. In the PBBI group, both PM and GM driven ATP synthesis remained higher compared to that driven by Succ and BHBM. The ATP synthesis between PM, Succ, and BHBM were significantly distinct amongst each other (PM = GM>Succ>BHBM, ^*^p < 0.05).

### Mitochondrial Dehydrogenase Enzyme Activities in PBBI

**Figure 5:**
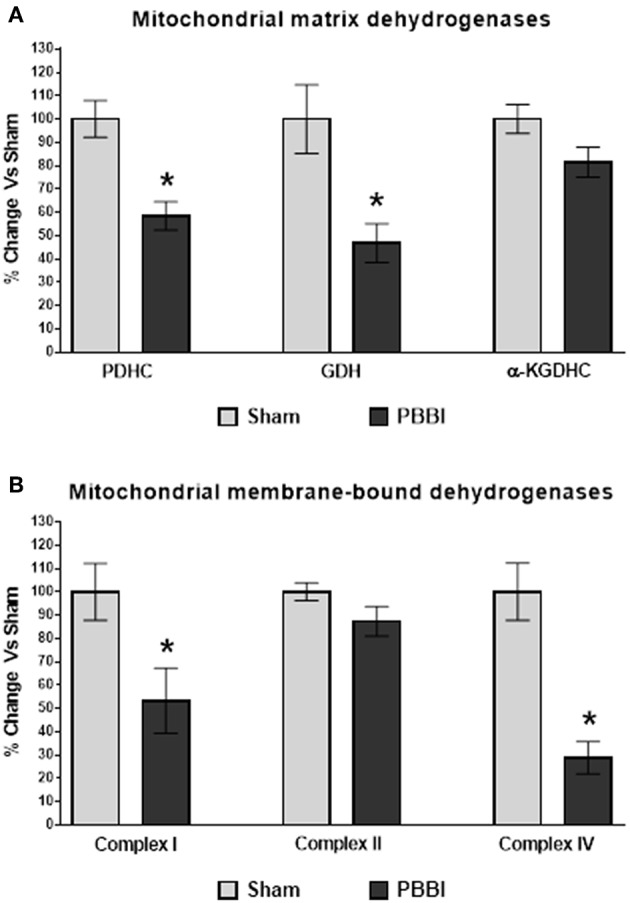
Decreased dehydrogenase activities following PBBI. **(A)** At 24 h post-PBBI, PDHC (42%, *p* < 0.05) and GDH (53%, *p* < 0.05) enzyme activities were significantly decreased; whereas no significant differences were noted in α-KGDHC activity between groups. **(B)** The Complex I (47%, ^*^p < 0.05) and Complex IV (81%, ^*^p < 0.05) enzyme activities of ETC were significantly impaired in the PBBI group; whereas no change in Complex II enzyme activity was observed between experimental groups. Bars represent group means ± SEM (*n* = 5 animals / group). ^*^p < 0.05 compared to sham injured group (unpaired *t*-test).

Mitochondrial matrix dehydrogenases, PDHC and GDH activities were significantly decreased compared to the sham group (42 and 53%, respectively, ^*^p < 0.05). No injury-specific differences were detected in the α-KGDHC activity between PBBI and sham groups (Figure 5A). In the PBBI group, mitochondrial membrane-bound dehydrogenases, Complex I and Complex IV enzyme activities were significantly decreased compared to the sham group (47 and 81%, respectively, ^*^p < 0.05); whereas Complex II activity was comparable to that in the sham group (Figure 5B).

### Decreased Coenzyme Contents in PBBI

**Figure 6:**
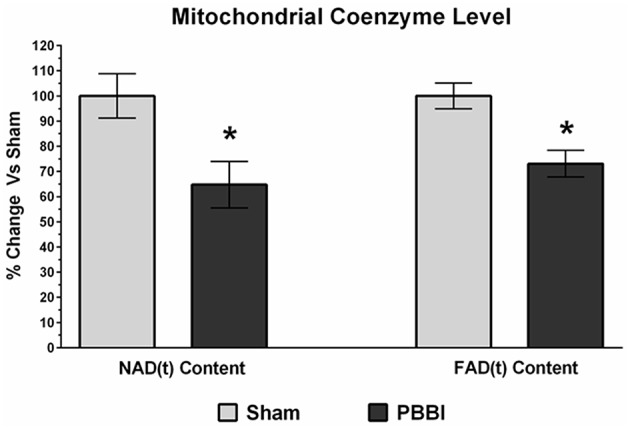
Decreased coenzyme contents following PBBI. At 24 h post-PBBI, significant reduction in mitochondrial coenzyme contents were detected. Both NAD_(t)_ (35%, *p* < 0.05) and FAD_(t)_ contents (27%, ^*^p < 0.05) were decreased significantly in the PBBI group (Unpaired *t*-test, ^*^p < 0.05). Bars represent group means ± SEM (*n* = 5 animals / group). ^*^p < 0.05 compared to sham injured group (unpaired *t*-test).

Mitochondrial coenzyme contents (i.e., NAD_(t)_ and FAD_(t)_) were quantitatively measured in PBBI and sham groups. In the PBBI group, both NAD_(t)_ (35%, ^*^p < 0.05) and FAD_(t)_ contents (27%, ^*^p < 0.05) were significantly decreased compared to the sham group (Figure 6).

## Discussion

The uninjured healthy brain preferentially utilizes glucose intermediates (vs. ketones) as the primary and obligated source of energy-rich metabolic fuels (35). Our previous research revealed significant alterations in metabolic responses between 30 min and 7 d post-PBBI, including osmotic stress, neurotransmitters imbalance, hyper-glycolysis, decreased TCA cycle anaplerotic metabolism, depletion of creatine stores, and enhanced complex lipid hydrolysis (36). We also detected altered glucose uptake and oxygen consumption in the injured cortex, including both the injury core and the peri-lesional area, at 2.5 h post-PBBI (37). In addition, using intracerebral microdialysis, we have reported an early energy metabolic dysfunction and the metabolic shift toward anaerobic glycolysis (indicated by high lactate to pyruvate ratio) within 3 h following PBBI (38).

The current study was designed to examine the acute brain mitochondrial bioenergetics dysfunction following PBBI based on our previous observations of the cerebral metabolomics alterations in this brain injury model. Results of the current study demonstrate mitochondrial bioenergetics failure evident at 24 h post-PBBI, which was likely due to a global declines in substrate utilization for energy production, including altered mitochondrial dehydrogenase activities and coenzyme contents. These results correspond with and build on previous work conducted in the CCI model of TBI (16, 34, 39–44). In the CCI model, the majority of published studies have utilized only pyruvate+malate (PM) as the energy substrates to evaluate Complex I driven mitochondrial respiration, in which the PM dependent mitochondrial ATP synthesis rates were significantly reduced within 30 min post-injury (39) and remain compromised up to 2–5 d post-injury (40–42). However, Xiong et al. reported that glutamate+malate (GM) dependent Complex I driven mitochondrial ATP synthesis was decreased between 1 h and 14 d post-injury (43). In the current study, we evaluated both PM and GM as metabolic substrates for complex I driven mitochondrial respiration in the PBBI model. Our results showed a reduction in both complex I driven ATP synthesis rates (State III) and uncoupling rates (State V) following PBBI (Figure 2). Notably, we had previously hypothesized that the reduction in PM driven ATP synthesis may be counterbalanced by the use of GM as an alternative metabolic substrate to produce ATP following PBBI, based early evidence that showed elevated extracellular glutamate levels following TBI (45–47). However, results from the current study showed a GM-dependent non-significant decrease trend in ATP synthesis following PBBI, which indirectly suggests that the glutamate substrate may not be an efficient alternative fuel for energy replenishment following PBBI.

We used two additional glucose and ketone intermediate substrates, succinate (Succ), and beta-hydroxybutyrate+malate (BHBM), to evaluate their bioenergetics capacity in the PBBI model (Figure 3). Both of these substrates support Complex II driven mitochondrial respiration for ATP synthesis, and thereby may bypass the Complex I driven respiration and partially circumvent the dependence on both PDHC and Complex I enzyme functions. It was reported that both PDHC and Complex I enzyme activities were significantly decreased at 3 h post-injury following CCI (34). To evaluate whether the Complex II driven respiration is also compromised, the Succ and BHBM dependent mitochondrial ATP synthesis rates were measured in isolated mitochondria following TBI. The results showed that both Succ and BHBM dependent ATP synthesis were significantly decreased following PBBI, which indicates that Complex II driven energy production were significantly compromised. Together, a significant decline in both Complex I and II driven ATP synthesis rates indicated global metabolic depression following PBBI. As such, this global energy crisis might potentially be mitigated using energy enhancers as a therapeutic treatment for acute TBI.

In support of this hypothesis, recent efforts were carried out to potentiate brain energy metabolism using alternative energy substrates in TBI patients. In one such study, Jalloh et al. reported that the ^13^C-labeled succinate (12 mmol/L for 24 h) perfused via intracerebral catheter decreased the lactate/pyruvate ratio measured in microdialysate samples, and improved glucose and glutamate utilization, thereby favoring aerobic glycolysis through metabolic flux into the TCA cycle in nine TBI patients with Glasgow Coma Scale ≤ 8 (48). These results provide support for the use of TCA cycle products or alternative energy substrates for enhancing brain mitochondrial metabolism in TBI patients as previously suggested (49–56).

In the current study, substrate dependent ATP synthesis rates were compared among major energy intermediates (i.e., pyruvate, glutamate, succinate and β-hydroxybutyrate) to identify the mitochondria preferred energy substrates at 24 h following either sham or PBBI procedure (Figure 4). Under the sham condition, the ATP synthesis rate of PM, GM, and Succ were unchanged amongst each other and were all significantly higher than that of BHBM (PM = GM = Succ>BHBM). Similarly, Berg et al. observed that the uninjured healthy brain mitochondria preferred glucose intermediates over ketones as energy substitutes and the metabolic responses were tissue-specific (35). In the current study, it was postulated that the metabolic substrates preference for energy production may have changed due to incurred metabolic stress following PBBI. Interestingly, our results showed that under the PBBI condition, PM and GM remained as preferred substrates for the injured brain, while the ketone BHBM utilization remained as the lowest compared to other glucose intermediates (PM = GM>Succ>BHBM). This is the first time that global metabolic substrate utilization pattern for ATP synthesis was evaluated in *ex vivo* condition following TBI. Overall, our results showed that glucose intermediate substrate utilization was declined following injury and ketone utilization remained the lowest when tested in *ex vivo* condition using isolated mitochondria from the PBBI brains.

All four metabolic substrates tested here for ATP synthesis showed energy deficits at 24 h post-PBBI, suggesting that therapeutic treatments should be initiated as early as possible to mitigate secondary injury responses of energy crisis following PBBI. Additionally, it may possible that these mitochondrial energy dysfunctions persist upto 2–5 d post-PBBI, as observed in the CCI model (40, 41). Similar to the current energy deficits observed at the injury core and peri-lesional brain regions (i.e., frontal cortex and striatum), the other brain regions which are distant to the injury site may show mitochondrial bioenergetics impairments. Therefore, a comprehensive post-injury time-course analysis of mitochondrial bioenergetics from different brain regions are warranted to better understand the brain region-specific injury responses following PBBI.

Several mitochondrial proteins involved in the cellular bioenergetics displayed oxidative modification following TBI. A proteomics study carried out by Opii et al. observed that mitochondrial dehydrogenase activities were reduced at 3 h post-CCI and several mitochondrial proteins displayed oxidative damage following TBI (34). In the current study, we measured mitochondrial dehydrogenase activities at 24 h post-PBBI (Figure 5). The mitochondrial matrix dehydrogenase (i.e., PDHC and GDH) activities were significantly reduced following PBBI, whereas the α-KGDHC activity was comparable between the sham and PBBI groups. The reduced PDHC and GDH enzyme activities may affect the PM and GM dependent ATP synthesis rates in the PBBI group as discussed previously. Moreover, the mitochondrial membrane-bound dehydrogenase Complex I and IV activities were significantly decreased after PBBI. In contrast, Complex II enzyme activity was not affected following PBBI. Originally, it was postulated that mitochondrial dehydrogenase activities would drop significantly due to the observed global decline in energy metabolism indicated by ATP synthesis and coenzyme (NAD_t_ and FAD_t_) contents following PBBI. However, it is counterintuitive that both α-KGDHC and Complex II enzyme activities remained unchanged despite of their cofactors' levels were significantly lower following PBBI. Overall, these data suggest the differential susceptibility of mitochondrial dehydrogenase activities to secondary injury, possibly due to the divergent redox-state sensitivity within their structures, ultimately leading to altered substrates oxidation, metabolic suppression, and energy depletion following injury. However, this hypothesis warrants further evaluation to confirm the causative effects of mitochondrial enzyme oxidation on the post-injury energy metabolism.

The use of Complex II driven energy substrates such as succinate or ketones, which oxidation feed electrons through Complex II, would be a good choice as “alternative biofuels,” given that they bypass the enzymatic dysfunction of both PDHC and Complex I in OXPHOS following PBBI. In the literature, pro-drugs that serve as alternative-energy substrates have been evaluated, glyceryl triacetate, or acetyl L-carnitine, which may bypass mitochondrial PDHC deficiency following brain and spinal cord injuries (12, 54, 55, 57–59). Similarly, decreases in PDHC and Complex I enzyme activities following PBBI observed here may be bypassed by the utilization of complex II driven alternative energy fuels for severe penetrating brain trauma. Note that the Complex IV enzyme activity was severely reduced following PBBI, consistent with previous findings in the CCI model (34). As Complex IV is the terminal enzyme complex of the ETC oxidative phosphorylation, any therapeutic intervention discussed above designed to bypass PDHC and Complex I enzymes may not be exclusively efficient to overcome complex IV deficiency. Therefore, drug intervention that can prevent or delay damage to Complex IV enzyme may prove beneficial for therapeutic purposes in combination with alternative biofuels/drugs to increase ATP synthesis following injury. Comprehensively, the current study has evaluated several mitochondrial targets, which can be bypassed / protected individually or in combination in the future using therapeutic interventions to alleviate metabolic depression following severe penetrating TBI.

In the current study, we measured both NAD_(t)_ and FAD_(t)_ contents using biochemical assays. These cofactors play essential roles in many cellular and mitochondria specific metabolic reactions by acting as electron (e^−^) and proton (H^+^) donors for substrate oxidation. In the mitochondrial ETC chain, the NADH transfers electrons to Complex I, whereas the FAD is a prosthetic group of Complex II which receives electrons from succinate, thereby bypassing Complex I for ATP synthesis. Our data presented here (Figure 6) showed a significant decline in both NAD_(t)_ and FAD_(t)_ contents following PBBI, which indicated that the capability to carry out efficient electron and proton transfers for ATP synthesis remained limited. Therefore, therapeutics that target replenishment of NAD_(t)_ and FAD_(t)_ may be useful in combination with Complex IV agonists to enhance energy efficiency following severe TBI. Efforts toward evaluating NAD and its precursors, nicotinamide and nicotinic acid, as neuroprotective agents for TBI and ischemic brain injury have shown some improvement in behavioral functions (60–68). However, more rigorous efforts are needed to validate bioenergetics and neuroprotective efficacy of both NAD and FAD precursors as a treatment for TBI.

In summary, our study provided a comprehensive evaluation of mitochondrial dysfunction at 24 h following PBBI. We observed glucose or ketone intermediate substrates mediated decline in ATP synthesis following PBBI. Additionally, mitochondrial dehydrogenase activities and coenzyme contents were significantly decreased following PBBI. While additional experiments are warranted to provide a comprehensive time-course and injury severity profile of mitochondrial bioenergetics in the PBBI model, the results of the current study provide a basis for the use of “alternative biofuels” for achieving higher ATP production following severe penetrating brain trauma.

## Ethics Statement

All experimental procedures were approved by the Institutional Animal Care and Use Committee (IACUC) at Walter Reed Army Institute of Research (WRAIR). Animal studies were conducted in compliance with the Animal Welfare Act, the Guide for the Care and Use of Laboratory Animals (National Research Council), and other federal statutes and regulations relating to animals and experiments involving animals.

## Author Contributions

JP contributed in literature review, research experiments, data analysis, and manuscript writing. XY and WF provided technical assistance. LL, YD-B, JG, and DS participated in experimental design and manuscript writing.

### Conflict of Interest Statement

The authors declare that the research was conducted in the absence of any commercial or financial relationships that could be construed as a potential conflict of interest.

## Abbreviations

ATP: Adenosine 5′-Triphosphate
α-KGDHC: alpha-Ketoglutarate Dehydrogenase Complex
ETC: Electron Transport Chain
FAD: Flavin Adenine Dinucleotide (oxidized)
FADH_2_: Flavin Adenine Dinucleotide (reduced)
FAD_(t)_: Flavin Adenine Dinucleotide (total content)
GDH: Glutamate Dehydrogenase
NAD: Nicotinamide Adenine Dinucleotide (oxidized)
NADH: Nicotinamide Adenine Dinucleotide (reduced)
NAD_(t)_: Nicotinamide Adenine Dinucleotide (total content)
OXPHOS: Oxidative Phosphorylation
PBBI: Penetrating Ballistic-Like Brain Injury
PDHC: Pyruvate Dehydrogenase Complex
TBI: Traumatic Brain Injury
TCA: Tricarboxylic Acid Cycle.
